# Tuberculosis Patients’ Serum Extracellular Vesicles Induce Relevant Immune Responses for Initial Defense Against BCG in Mice

**DOI:** 10.3390/microorganisms13071524

**Published:** 2025-06-29

**Authors:** Wenzhao Xu, Yue Hou, Jingfang Zhang, Tingming Cao, Guangming Dai, Wenjing Wang, Na Tian, Dingyi Liu, Hongqian Chu, Hong Sun, Zhaogang Sun

**Affiliations:** Beijing Chest Hospital, Capital Medical University & Beijing Tuberculosis and Thoracic Tumor Research Institute, Translational Medicine Center, Beijing 101149, China; xwz_6729@163.com (W.X.); hou9702@126.com (Y.H.); zhangjingfang@bjxkyy.cn (J.Z.); tmcao@sina.com (T.C.); dai0472gm@163.com (G.D.); wwj28107@163.com (W.W.); tianna1998@163.com (N.T.); ldy20221@163.com (D.L.); chuhongqian@bjxkyy.cn (H.C.)

**Keywords:** *Mycobacterium bovis bacillus Calmette Guérin*, extracellular vesicles, serum, immunity, mouse

## Abstract

Extracellular vesicles (EVs) can be distributed in various bodily fluids, such as serum and urine, and play an essential role in immune regulation, substance transport, and other aspects. Tuberculosis (TB) is an infectious disease caused by *Mycobacterium tuberculosis* (*Mtb*), which places a tremendous burden on public health prevention and control within society. Researchers are committed to developing various diagnoses and treatment plans to eliminate TB effectively. The results of some studies conducted to date demonstrate that the serum EVs of TB patients, which carry components related to *Mtb*, can be used as relevant markers for TB detection and improve diagnostic efficiency. However, no relevant reports exist on the particular physiological functions such EVs perform, thus warranting further exploration. In this study, we collected serum EVs from both healthy individuals and TB patients. After identifying the morphology, concentration, and expression of classic markers (CD63, CD81, and CD9) of EVs, we explored their physiological functions at the cellular level and their physiological functions and effects on BCG colonization in the lungs at the mouse level. It was found that EVs were abundant in TB patients and healthy individuals, and the number of CD63 and CD9 markers co-expressed on the surface of serum EVs in healthy individuals was greater than that in TB patients. Serum EVs in patients with TB can stimulate cells to secrete more immune cytokines, such as TNF-α and IL-6, compared with those in healthy individuals; induce an increase in the M1/M2 ratio of macrophages in the peripheral blood mononuclear cells of mice; and inhibit the colonization of *Mycobacterium bovis bacillus Calmette Guérin* (BCG) in the lungs of mice. In addition, they can inhibit the occurrence of inflammatory responses in the lung tissue of mice. The above results suggest that serum EVs in TB patients may exert their physiological function by regulating immune responses. This finding also indicates that exploring serum EVs in TB patients with regard to their physiological functions shows excellent potential.

## 1. Introduction

Tuberculosis (TB) remains one of the deadliest infectious diseases, placing tremendous pressure on the World Health Organization with regard to prevention and control [1,2]. TB is a respiratory disease caused by *Mycobacterium tuberculosis* (*Mtb*) and is most commonly found in the lungs [3,4]. Research has shown that *Mtb* has the ability to both activate and escape host immunity; in one instance, it can activate the host’s production of cytokines such as IL-6 and TNF-α to regulate the immune response to inhibit the survival of *Mtb* [5,6]; conversely, it can evade the host immune response by modulating certain intracellular signaling pathways through *Mtb*-related molecules in order to survive in the host [7,8]. The main active cells of *Mtb* are macrophages [9], and they interact closely with macrophages in the host. Macrophages are important participants in the host’s immune response [10]. Macrophages can be classified into M1 type and M2 type. Of the two types, M1-type macrophages are also known as classically activated macrophages and play a pro-inflammatory role; in contrast, M2-type macrophages are referred to as optionally activated macrophages [11,12]. By exerting anti-inflammatory/profibrotic effects, M1-type macrophages play an important role in host defense by expressing pro-inflammatory and antibacterial molecules, such as interleukin-6 (IL-6), tumor necrosis factor-α (TNF-α), etc. [12,13]. M2 macrophages aid in maintaining tissue homeostasis and controlling inflammation by expressing anti-inflammatory cytokines at high levels, such as interleukin-10 (IL-10), C-C motif chemokine ligand 1 (CCL1), etc. [13,14]. Macrophages in the host can be polarized via *Mtb* stimulation, and this polarization response is crucial for the immune response of *Mtb* [15]. Research has shown that during acute infection with *Mtb*, macrophages can polarize to type M1 and activate multiple cellular signaling pathways. For example, *Mtb* can stimulate the TLR2/TLR4 signaling pathway [16,17] and secrete cytokines such as IL-6 and TNF-α to clear *Mtb* [18,19,20]. In the same manner, M2-type macrophages can play a key role in assisting *Mtb* in generating immune escape responses within the host [21,22], through specific components secreted by *Mtb*. For example, the PI3K/Akt1/mTOR signaling pathway is regulated by Rv1987, which is expressed explicitly in pathogenic mycobacteria [23], and cytokines such as interleukin-4 (IL-4) and C-C motif chemokine ligand 22 (CCL22) are expressed to control inflammation and promote the growth and survival of *Mtb* in the host [21,24,25]. It can be concluded from the above findings that M1 and M2 macrophages are crucial for the survival of *Mtb* in the host.

Not only can *Mtb* modulate host immune responses, but some extracellular vesicles (EVs) also carry *Mtb*-related molecules and possess the ability to modulate host-related physiological functions [26,27]. These molecules include EVs secreted by *Mtb* itself [28] and those secreted after *Mtb* infection of cells [29]. EVs are membrane-structured vesicles secreted by cells, bacteria, etc., that can contain a variety of substances, such as proteins, lipids, nucleic acids, etc [30,31]., and they exert specific physiological functions. EVs can be present in cell culture supernatants, urine, serum, plasma, and many other bodily fluids [32], making their presence extremely widespread. The researchers of studies on EVs in *Mtb* bodily fluids have mainly focused on serum [33], plasma [34], urine [35], pleural fluid [36], etc. Most of these studies concern the types of *Mtb*-related molecules contained in the EVs of bodily fluids and the screening of molecular markers for the diagnosis of TB, with less attention paid to the physiological functions of EVs.

Due to the advantages presented by blood and urine samples, which are simple and easy to obtain, and the fact that blood contains richer individual information than urine and serum is easier to process than plasma, currently, the information obtained regarding the physiological functions of serum EVs in TB patients is limited. In light of the above findings, in this study, EVs from the serum of TB patients were selected to investigate their related physiological functions. In this study, for EVs collected from the serum of healthy individuals and TB patients (based on the nomenclature of the International Society for Extracellular Vesicles (ISEV) [37], we use the term EV to represent all lipid bilayer particles contained in the serum mentioned herein whose diameters are less than 200 nm), the experimental procedure is shown in Figure S1. The enriched EVs were characterized via transmission electron microscopy (TEM), nanoparticle tracking analysis (NTA), and an exosome detection chip. The toxicity and phagocytosis effects of EVs on cells were further verified using CCK-8 and confocal immunofluorescence microscopy, in addition to it being verified through qPCR that TB patients’ serum EVs can stimulate cells to produce the cytokines IL-6 and TNF-α. Mice were stimulated with TB patients’ serum EVs and then with the *Mycobacterium bovis bacillus Calmette Guérin* (BCG) vaccine, and a decrease in the lung colonization of BCG could be observed in colony forming units (CFUs). Hematoxylin eosin (H&E) staining revealed that the inflammatory condition of the lung tissue was weakened. The flow analysis of macrophages extracted from the venous blood of mice revealed an increase in the number of M1 macrophage cells and an increase in the M1/M2 ratio. The above results suggest that the serum EVs of TB patients have a certain potential in regulating the host immune response and inhibiting the survival of bacteria in vivo.

## 2. Materials and Methods

### 2.1. Reagents

In this study, fetal bovine serum (FBS) (F101-01), a FastPure Complex Tissue/Cell Total RNA Isolation Kit (RC113-01), and a BCA Protein Quantification Kit (E112-01) were obtained from Vazyme (Nanjing, China). DMEM (319-005-CL) and RPMI1640 (350-000-CL) culture medium were obtained from WISENT (Nanjing, China). Tissue fixative (G1101) and SweScript All-in-One RT SuperMix for qPCR (G3337-50) were procured from Servicebio (Wuhan, China). An SYBR^®^ Fast Universal qPCR Kit (F0107) was acquired from Forscience (Beijing, China). CCK-8 (C6005) and ProtLytic Protease Inhibitor Cocktail (P001) were obtained from NCM (Suzhou, China). Cell Staining Buffer (420201), Brilliant Violet 421™ anti-mouse CD163 (155309), Brilliant Violet 650™ anti-mouse F4/80 (123149), APC/Cyanine7 anti-mouse CD45 Antibody (103115), and Brilliant Violet 605™ anti-mouse CD80 (104729) were acquired from Biolegend (San Diego, CA, USA). An ExoView comprehensive characterization kit for exosomes (EV-TETRA-C) was obtained from Quantum Design (San Diego, CA, USA). An Exosupur^®^ EV Re-purification Pocket Column (CTE-ES9016) was obtained from ECHO BIOTECH (Beijing, China). Lastly, a Manual Exosome Isolation kit EIQ3-01001 (for serum) was acquired from H.Wayen Biotechnologies (Shanghai, China).

### 2.2. Cell Lines, Bacterial Strains, and Animals

The RAW264.7 cells and CT26 cells (ATCC) used in this study were preserved in our laboratory and cultured in DMEM and RPMI 1640 medium containing 10% FBS at 37 °C and 5% CO_2_, respectively.

RAW 264.7: RAW 264.7 is an adherent cell line isolated from a mouse tumor that was induced by Abelson murine leukemia virus. This cell line, with macrophage differentiation, can be used in oxidative stress, inflammatory, and antibacterial activity studies. RAW 264.7 cells are tumorigenic and are widely used in cancer and drug development research.

CT26.WT: CT26 cells are undifferentiated colon cancer cell lines induced by N-nitroso-n-methylcarbamate (NNMU). CT26.WT cells possess homozygous Kras mutations (p.G12D) and homozygous deletions (Cdkn2a). In these cells, proliferation and stem cell markers (such as Top2a, Birc5, Cldn6, and Mki67) are highly expressed, whereas differentiation and top-crypt markers (such as Muc2, Ms4a8a, and Epcam) are absent. The effects of this cell line on tumor growth, metastasis, cytotoxicity, and apoptosis can be assessed. These cells can be used as a model for testing immunotherapy procedures and studying the host immune response.

BCG was derived from a strain from the Beijing Tuberculosis and Thoracic Tumor Research Institute and was cultured in 7H9 liquid medium (271310, BD, New York, NY, USA) containing 10% OADC (212352, BD, NY, USA) and cultured for approximately one week before further experimentation at 37 °C.

The mice used in this study were female 6-week-old Balb/c mice acquired from Vital River Laboratory Animal Technology Co., Ltd (Beijing, China). All mice were transferred to the specialized laboratory after confirmation that they were free of specific pathogens. This study was approved by the Experimental Animal Welfare Ethics Committee, Beijing Chest Hospital, which is affiliated with Capital Medical University (approval code: XK2024-121 and 7 June 2024).

### 2.3. Extraction of Serum EVs

Serum from patients diagnosed with pulmonary TB and healthy individuals not suffering from TB was collected (Table S1). The serum collection procedure was approved by the Ethics Committee of Beijing Chest Hospital, which is affiliated with Capital Medical University (approval code: 2022-18 and 16 March 2022). Serum EVs were separated using an exosome separation kit (EIQ3-01001, H.Wayen Biotechnologies, Shanghai, China) according to the manufacturer’s instructions. After extraction, the EVs were suspended in 200 μL PBS and quantitatively determined with a BCA protein quantitative kit (WB6501, NCM, Suzhou, China).

### 2.4. Transmission Electron Microscope (TEM) Analysis

The EV samples were diluted 1:50 with PBS (G4202, Servicebio, Wuhan, China); the diluted samples were mixed well and 15 μL was aspirated on a copper mesh (FF150, HEAD, Beijing, China) and left for 1 min. Next, 15 μL of 2% dihydroxyuranium acetate staining solution (02624-AB, SPI, South San Francisco, CA, USA) was added, and the surface of the copper mesh was rinsed gently with ddH_2_O and air-dried; thereafter, the samples were observed with a transmission electron microscope (HT7800, HITACHI, Tokyo, Japan), and the corresponding images were recorded.

### 2.5. Nanoparticle Tracking Analysis (NTA)

The EV samples were diluted 1:100 using PBS (G4202, Servicebio, Wuhan, China), and the diluted samples were mixed and observed using nanoparticle tracking analysis (ZetaView, Particle Metrix, Meerbusch, Germany), and the corresponding data were recorded.

### 2.6. EV Microarray Assay

The EV samples were diluted 1:50 using PBS (G4202, Servicebio, Wuhan, China), and the diluted samples were mixed. The EV samples were co-incubated with microarrays according to the manufacturer’s instructions, and the NanoView Fully automatic Exosome fluorescence Detection System (ExoView^®^ R200, QUANTUM, San Jose, CA, USA) was used to collect data.

### 2.7. Cell Activity Assay

EV concentrations were set at 5 μg/mL, 10 μg/mL, 50 μg/mL, and 100 μg/mL and incubated with CT26 cells and RAW264.7 cells in a 96-well plate (CCP-96H, Servicebio, Wuhan, China) for 24 h at 37 °C. The culture supernatant was removed and 10 μL CCK-8 (C6005, NCM, Suzhou, China) reagent was added to each well; following incubation at 37 °C with 5% CO_2_ for 15 min, absorbance was detected at OD450 nm using a Multiskan™ FC Microplate Photometer (1410101, Thermo fisher, Waltham, MA, USA).

### 2.8. qPCR

Next, 5 μg/mL EVs were incubated with CT26 or RAW264.7 cells for 4 h. After the culture supernatant had been discarded, it was replaced with fresh medium at 37 °C and 5% CO_2_ and incubated for 24 h. Thereafter, the cells were absorbed for RNA extraction using a FastPure Complex Tissue/Cell Total RNA Isolation Kit (RC113-01, Vazyme, Nanjing, China) according to the manufacturer’s instructions. A SweScript All-in-One RT SuperMix for qPCR kit (G3337-50, Servicebio, Wuhan, China) was used to reverse-transcribe RNA to cDNA. The primers listed in Table S2 (primer sequences are referenced from [38,39]) were synthesized through Sangon Biotech (Shanghai) Co., Ltd (Shanghai, China), and configured into a mixture using the SYBR^®^ Fast Universal qPCR kit (F0107, Forscience, Beijing, China). The qPCR was performed and data were collected on an ABI real-time fluorescence quantitative PCR instrument (7500, Thermo fisher, MA, USA).

### 2.9. FACS

Following stimulation of the mice with EVs and BCG, venous blood was collected in EDTA anticoagulation tubes (367525, BD, NY, USA) at 4 weeks, and lymphocytes were extracted from the blood using a mouse peripheral blood single cell extraction kit (P6340, Solarbio, Beijing, China). Antibody staining was performed on mouse lymphocyte samples based on the specified concentrations, and after avoiding light at 4 °C for 45 min, the stained cells were centrifuged at 500× *g* and 4 °C for 5 min. Thereafter, the stained cells were rinsed once and centrifuged at 500× *g* and 4 °C for 5 min. Next, 400 μL cell staining buffer (420201, Biolegend, CA, USA) was used to resuspend the cells, and detection was performed with a BD Fortessa analytical flow cytometer (LSRFortessa, BD, New York, NY, USA).

### 2.10. EV Stimulation and BCG Infection in Mice

Based on the results of BCA quantification, healthy individuals’ serum EVs and TB patients’ serum EVs were adjusted to 100 μg per mouse [40]. Thereafter, both types of EVs were injected into mice at a volume of 100 μL through the tail vein, and 100 μL PBS was injected into the control group. One week later, 1 × 10^7^ CFU/mL of BCG 100 μL per mouse was injected into all of the mice through the tail vein, and the mice were euthanized at 4 weeks.

### 2.11. H&E

As noted above, the mice stimulated by EVs and BCG were euthanized at 4 weeks. The heart, liver, spleen, kidneys, and lungs of the mice were removed and fixed in tissue fixation solution (G1101, Servicebio, Wuhan, China). After fixation, HE staining was performed and an optical microscope was used to observe the samples (BX53M, Olympus, Tokyo, Japan).

### 2.12. Immunofluorescence (IF)

The EVs were incubated with 5 μM Dil (C1036, Beyotime, Shanghai, China) at room temperature for 20 min, centrifuged at 120,000× *g* at 4 °C for 1 h, and then centrifuged with 10 mL PBS for 1 h and resuspended with 100 μL PBS (G4202, Servicebio, Wuhan, China). The centrifuged EVs were incubated with CT26 or RAW264.7 cells for 4 h. After the culture supernatant had been discarded, fresh medium was added to the cells at 37 °C and 5% CO_2_ for 12 h. The cells were fixed with 4% paraformaldehyde (P0099, Beyotime, Shanghai, China) at room temperature for 15 min. The nuclei were stained with 1 μg/mL Hoechst 33342 (C1022, Beyotime, Shanghai, China) at room temperature for 15 min, and immunofluorescence images were taken using a confocal microscope (FV100, Leica, Wetzlar, Germany) at 405 nm and 555 nm.

### 2.13. Statistical Analysis

We utilized NanoView 1.0 software to analyze the results of the exosome detection chip. The data detailed in this paper were statistically analyzed using GraphPad Prism 8.0 and are presented as the mean and standard error (mean ± SD). Analysis of variance was used to compare the differences among the groups. *p* < 0.05 was considered statistically significant. (* *p* < 0.05; ** *p* < 0.01; ns: no significance.)

## 3. Results

### 3.1. Identification of EVs in the Sera of Healthy Individuals and TB Patients

**Figure 1:**
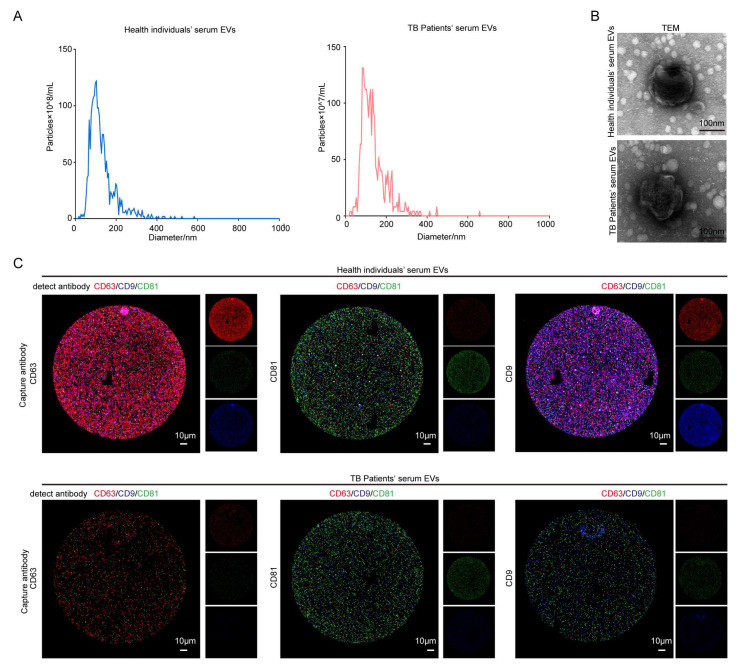
The identification of serum from healthy individuals and TB patients. (**A**) NTA analysis aided in the identification of the number and size of the EVs. (**B**) The morphology of EVs was observed via TEM (upper right). Scale bar: 100 nm. (**C**) An exosome detection chip was used to detect CD63, CD9, and CD81 in serum EVs. After incubating the EVs with the chip, the fluorescently labeled CD63, CD9, and CD81 antibodies were continuously incubated on the chip—red: CD63; green: CD81; blue: CD9. Left CD63: Using CD63 as the capture antibody, the fluorescence expressions of CD63, CD9, and CD81 were detected. All three fluorescence expressions were present, and the expression of red fluorescence and blue fluorescence in the healthy individuals’ serum EVs group was higher than that in the patient group, indicating that CD63, CD9, and CD81 in both groups of EVs were expressed as expected. The expressions of CD63 and CD9 in the healthy individuals’ serum EVs group were higher than those in the patient group. Intermediate CD81: CD81 is a capture antibody. The fluorescence expressions of CD63, CD9, and CD81 were detected. All three fluorescence expressions were present, and the green fluorescence expression in the healthy individuals’ serum EVs group was higher than that in the TB patient group, indicating that CD63, CD9, and CD81 in both groups of EVs were expressed as expected. The expression of CD81 in the healthy individuals’ serum EVs group was higher than that in the patient group. CD9 is the capture antibody illustrated on the right-hand side. The fluorescence expressions of CD63, CD9, and CD81 were detected. All three fluorescence expressions were present. Moreover, the expressions of red fluorescence and blue fluorescence in the healthy individuals’ serum EVs group were higher than those in the TB patient group, indicating that CD63, CD9, and CD81 in both groups of EVs were expressed as expected, and the expression of CD63 and CD9 in the serum EVs group of healthy individuals was higher than those in the patient group. Scale bar: 10 μm.

The results of a number of studies have shown that serum contains a large number of EVs [41]. In this study, three serum samples from healthy individuals and three serum samples from TB patients were collected for the extraction of EVs, which were subsequently characterized via NTA and TEM. The NTA results demonstrated that the two types of EVs were similar in size, with both of them measuring around 100 nm, in accordance with the standard size of EVs. The absolute number of serum EVs from TB patients was roughly 10^10^, which was identical to the number of serum EVs obtained from healthy individuals, 10^11^ (Figure 1A); the average diameters of serum EVs in healthy individuals and TB patients were 129.2 nm and 136.8 nm, respectively. Regarding serum EVs of healthy individuals, 90% of the particles had a particle size of less than 202.6 nm, and 10% had a particle size greater than 202.6 nm. Regarding serum EVs of TB patients, 90% of particles had a particle size of less than 217.6 nm, and 10% had a particle size greater than 217.6 nm. The diameters of serum EVs in the two groups were similar, with high quality noted (Table S3). The TEM results demonstrated that the EVs were rounded and wrinkled, conforming to the standard morphology of EVs (Figure 1B). Both groups of EVs contained the EV-specific markers CD63, CD9, and CD81 (Figure 1C), which are commonly used as markers for EV identification; of note, these markers are the most commonly used surface markers of EVs. They all possess four transmembrane domains and are, therefore, also called tetraspanins. They belong to the tetraspanin transmembrane protein family and play a crucial role in cell membrane tissue [42,43,44]. They regulate various cellular processes, such as the transport of their direct molecular chaperones and the compartmentalization function of the membrane region [45,46,47]. CD9 and CD81 are closely related tetra transmembrane proteins, sharing 45% of their identity and playing similar roles in cell fusion. Both CD9 and CD81 are directly associated with two related Ig domain proteins, CD9P-1/EWI-F (encoded by the PTGFRN gene) and EWI-2 (encoded by the IGSF8 gene) [47,48,49,50], and they also regulate several fusion processes [51,52,53]. Evidence from recent studies demonstrates that more than one CD9 molecule is present in EVs, and co-localization between CD9 or CD63 or CD81 and CD63 or CD81 is very common. The researchers of recent EV studies emphasize the specific heterogeneity of classical EV markers (such as tetra transmembrane proteins) in EV populations released by different cell types [42]. In this study, we detected EVs through the exosome detection chip with fluorescent antibodies against CD63, CD9, and CD81. The above results indicate that the two groups of EVs were successfully extracted.

**Figure 2:**
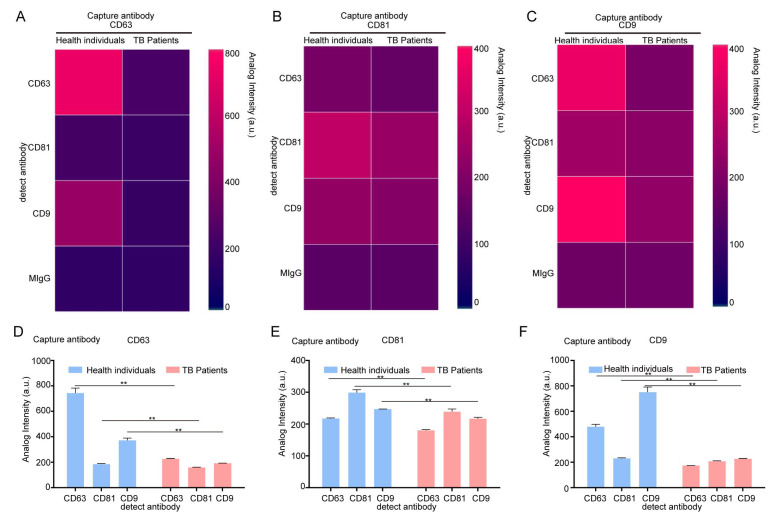
The identification of serum from healthy individuals and TB patients using an exosome detection chip. (**A**–**C**) The fluorescence intensity of serum EVs was analyzed with an exosome analysis chip heat map. CD63 was the capture antibody used to examine the fluorescence expression of CD63, CD9, and CD81 (**A**); CD81 was the capture antibody used to examine the fluorescence expression of CD63, CD9, and CD81 (**B**); and CD9 was the capture antibody used to examine the fluorescence expression of CD63, CD9, and CD81 (**C**). (**D**–**F**) The fluorescence expression of serum EVs was analyzed using an exosome analysis chip. CD63 was used as a capture antibody to examine the fluorescence expression of CD63, CD9, and CD81 (**D**); CD81 was used as a capture antibody to examine the fluorescence expression of CD63, CD9, and CD81 (**E**); and CD9 was used to detect the fluorescence expression of CD63, CD9, and CD81 (**F**). The experiment was independently repeated three times in each group (n = 3); ** *p* < 0.01.

When CD63 was used as the capture antibody, the fluorescence intensities of CD63 and CD9 in the TB patient group were lower than that those in the healthy group. When CD81 was used as the capture antibody, the fluorescence intensity of CD81 in serum EVs in the TB patient group was lower than that in the healthy group. When CD9 was used as a capture antibody, the fluorescence intensity of CD63 and CD9 in the serum EVs of the TB patient group was lower than that of the healthy group (Figure 1C). Based on the fluorescence results presented in Figure 1C for fluorescence expression statistics, the statistical results of serum EVs in the healthy group and the patient group under different capture antibodies were obtained. The heat map (Figure 2A–C) and the bar chart (Figure 2D–F) are both different manifestations of the same statistical method. In the heat map, the more intense the red color, the higher the fluorescence intensity. When CD63 was used as the capture antibody, the fluorescence intensities of CD63 and CD9 in the TB patient group serum EVs were lower than those of the healthy group. When CD81 was used as a capture antibody, the fluorescence intensity of CD81 in serum EVs in the TB patient group was lower than that in the healthy group. When CD9 was used as the capture antibody, the fluorescence intensities of CD63 and CD9 in the serum EVs of the TB patient group were lower than those of the healthy group (Figure 2A–C). The expression results in the bar charts presented in Figure 2D–F are consistent with the analysis results of the fluorescence map and the heat map of fluorescence intensity. Moreover, when CD9 is used as the capture antibody, the fluorescence values of CD63 and CD9 in the healthy group are higher than those in the patient group, and there is a significant difference between the two groups. This finding indicates that the number of EVs co-expressing CD63 and CD9 in the serum group of TB patients is lower than that in the healthy group, which may be a relevant indicator for TB detection in the future.

### 3.2. Serum EVs from TB Patients Induce Increased Expression of Cytokines IL-6 and TNF-α in Cells

**Figure 3:**
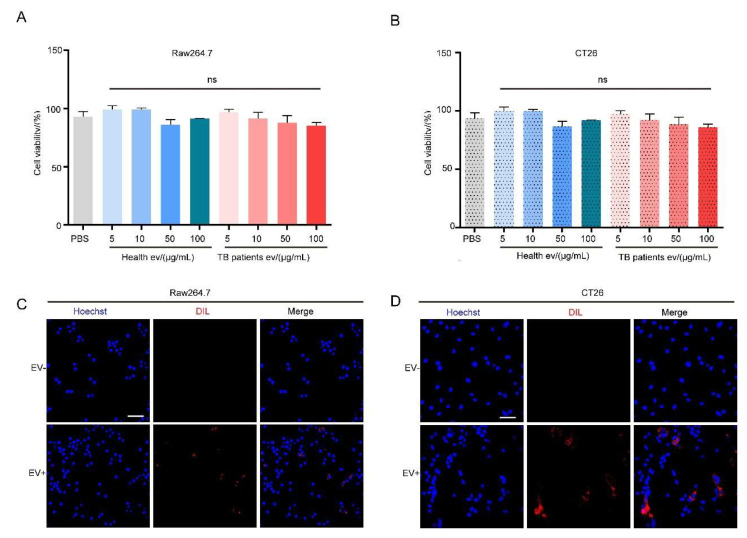
Serum EVs from TB patients cause no damage to the cells. (**A**,**B**) The effect of serum EVs on the activity of RAW264.7 (**A**) and CT26 (**B**) cells was detected using a CCK-8 assay. The experiment was independently repeated three times in each group, n = 3, ns: no significant difference. (**C**,**D**) Confocal immunofluorescence microscopy results of Dil-labeled serum EVs co-cultured with RAW264.7 (**C**) and CT26 (**D**) cells for 12 h. The cell nucleus (Hoechst) is marked in blue and EVs (Dil) are marked in red. Scale bar: 100 μm.

Research has shown that EVs can be endocytosed by cells, given the fact that they are cell-derived and possess a membrane structure similar to that of cells. In this study, we used EVs extracted from healthy individuals’ serum and TB patients’ serum quantified via BCA; the measured protein concentration was used as a basis to represent the number of EVs for subsequent assays [54], and the CCK-8 assay was performed following the co-incubation of EVs of different protein concentrations with CT26 or RAW264.7 cells. Our results demonstrated that RAW264.7 and CT26 cell activities were not disturbed at EV concentrations of 5, 10, 50, and 100 μg/mL (Figure 3A,B). In addition, we prepared Dil-labeled TB patients’ serum EVs, incubated them with RAW264.7 and CT26 cells for 12 h, respectively, and performed confocal immunofluorescence microscopy analysis. The results demonstrated that red fluorescence appeared in the cells represented by blue nuclei, indicating that both cells could endocytose serum EVs (Figure 3C,D).

**Figure 4:**
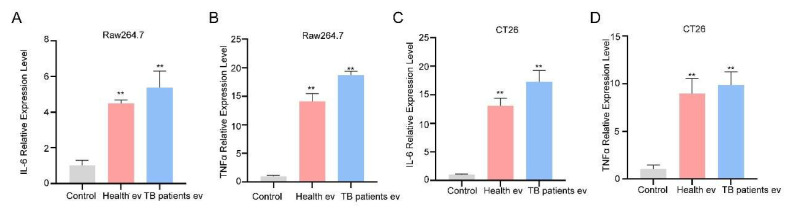
Serum EVs from TB patients induce the production of the cytokines IL-6 and TNF-α. (**A**,**B**) Serum EVs were incubated with RAW264.7 cells for 24 h, and cells were collected for the qPCR detection of IL-6 (**A**) and TNF-α (**B**) mRNA expression levels. (**C**,**D**) Serum EVs were incubated with CT26 for 24 h, and cells were collected for the qPCR detection of IL-6 (**C**) and TNF-α (**D**) mRNA expression levels. The experiment was independently repeated three times in each group (n = 3); ** *p* < 0.01.

The results of a number of studies have demonstrated that EVs not only act in a cargo transportation capacity but also exert functions such as regulating cell physiological activities [55]. The expression of cytokines IL-6 and TNF-α related to immune activation was detected at the RNA levels. The experimental results demonstrate that, compared with the unstimulated group, both healthy and TB patients’ serum EVs induced high expression of IL-6 and TNF-α in RAW264.7 and CT26 cells (Figure 4A–D); however, the TB patients’ serum group EVs induced higher expression of IL-6 and TNF-α in RAW264.7 and CT26 cells than those of the healthy group, suggesting that TB serum EVs can stimulate cells to produce more intense inflammatory responses.

### 3.3. Serum EVs from TB Patients Promote Macrophage Polarization to M1 Type in Mice In Vivo

**Figure 5:**
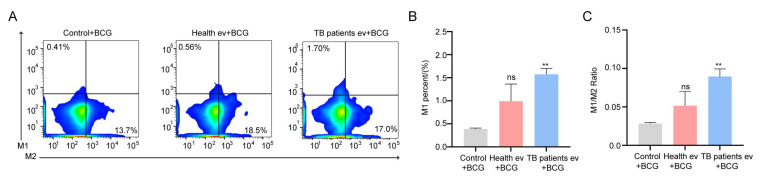
The polarization of macrophages in mice was influenced by the TB patients’ serum EVs. (**A**) Flow cytometry analysis of the macrophage horizontal of Balb/c mice. Control + BCG: BCG infection group; Health ev + BCG: mice stimulated with healthy individuals’ serum EVs alongside BCG infection; TB patients’ ev + BCG: mice stimulated with TB patients’ serum EVs alongside BCG infection. (**B**,**C**) Statistical data corresponding to flow cytometry results: M1 (**B**) and M1/M2 (**C**). The experiment was repeated three times in each group (n = 3); ** *p* < 0.01; ns: no significant difference. Control + BCG: BCG infection group; Health ev + BCG: mice stimulated with healthy individuals’ serum EVs alongside BCG infection; TB patients evs + BCG: mice stimulated with TB patients’ serum EVs alongside BCG infection.

At the cellular level, our results demonstrated that TB patients’ serum EVs can induce the strong expression of IL-6 and TNF-α in cells, which are closely related to the activation of immune cells [56,57], with the authors of some studies demonstrating that *Mtb* mainly interacts with host macrophages [58]; it is therefore assumed that TB patients’ serum EVs are also somehow associated with host macrophages. To better demonstrate the correlation more clearly at the animal level, in this study, the physiological functions of serum EVs from TB patients were explored to some extent at the mouse level. Firstly, we injected the same concentration of healthy individuals’ and TB patients’ serum EVs into the tail vein of our experimental mice to enable them to reach all parts of the body through blood circulation; thereafter, we injected the same concentration of BCG into the mice through the tail vein to ensure that the EVs and the BCG entered the body in the same manner, and the order of sequence was maintained throughout, so as to exclude unnecessary interference caused by injecting the BCG in another manner. After 4 weeks of collecting venous blood from the mice for flow cytometry analysis, we found that the number of macrophage M1 cells and the ratio of M1/M2 increased in the serum EVs of the TB patient group compared with those of the healthy individuals group and the BCG-infected group, and the number of M1 cells and the ratio of M1/M2 were greatest in the TB patient group, followed by the healthy individuals group, and lowest in the BCG-infected group (Figure 5A–C). This finding suggests that serum EVs from TB patients can stimulate macrophage polarization toward the M1 type, and their immunomodulatory effect is the strongest.

### 3.4. In Vivo Inhibition of BCG Growth in Mice Induced by Serum EVs Derived from TB Patients

**Figure 6:**
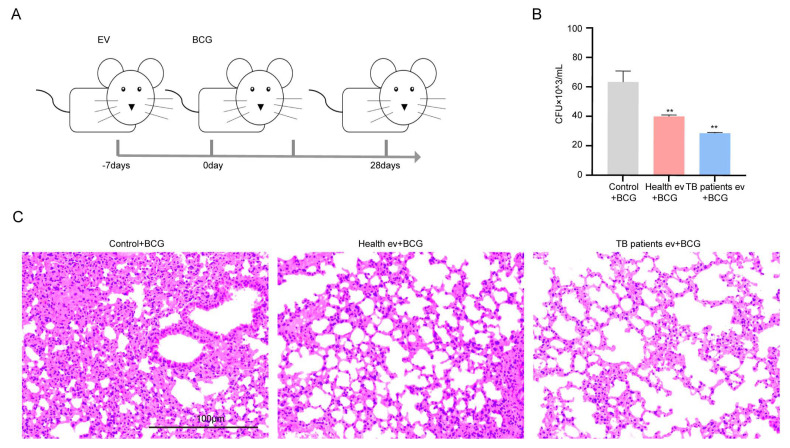
TB patients’ serum EVs inhibited the survival of BCG in mice. (**A**) Experimental process followed for the animal experiment: Balb/c mice were stimulated with serum EVs for one week and then stimulated with BCG, with sample collection performed based on the time starting point after BCG stimulation. (**B**) Balb/c mouse lung homogenate colony growth count (CFUs). Control + BCG: BCG infection group; Health ev + BCG: mice stimulated with healthy individuals’ serum EVs alongside BCG infection; TB patients ev + BCG: mice stimulated with TB patients’ serum EVs alongside BCG infection. The experiment was independently repeated three times in each group (n = 3); ** *p* < 0.01. (**C**) Lung H&E staining results of Balb/c mice; control + BCG: BCG infection group; Health ev + BCG: mice stimulated with healthy individuals’ serum EVs alongside BCG infection; TB patients ev + BCG: mice stimulated with TB patients’ serum EVs alongside BCG infection. Scale bar: 100 μm.

After detecting the in vivo immune level of mice modulated by serum EVs derived from TB patients, we collected the mice’s organs, namely, the heart, liver, spleen, lung, and kidney, to perform H&E staining and CFU estimation, with the experimental procedure outlined in Figure 6A. The results demonstrate that in the CFUs of mice lungs, the greatest number of bacteria was found in the simple BCG-infected group, followed by the healthy individuals’ serum EV-stimulated group, and the lowest number of BCGs was found in the TB patients’ serum EV-stimulated group (Figure 6B). The results of H&E staining demonstrated that the highest degree of inflammatory infiltration was found in the lung tissues of the simple BCG-infected group; in comparison, the degree of inflammatory infiltration decreased in the healthy individuals’ serum EV-stimulated group and even more so in the TB patients’ serum EV-stimulated group (Figure 6C). The degree of inflammatory infiltration in the spleen followed the same trend as that observed in the lung tissue (Figure S2); in comparison, the heart, liver, and kidney were not significantly impacted (Figure S3). The above results suggest that serum EVs from TB patients have an inhibitory effect on the growth activity of BCG in the lungs of mice.

## 4. Discussion

Exosome microarray detection technology is a method used for the detection of EVs that has emerged in recent years; it captures exosomes in samples using capture antibodies and enables the detection of relevant protein markers against them [59]. In this paper, we determined the preparation of the EVs by detecting the standard EVs protein markers CD63, CD81, and CD9 in serum EVs from TB patients, and we also determined the fluorescence expression of CD63, CD81, and CD9 in the TB patient group by analyzing the fluorescence intensity of them when CD63, CD81, and CD9 were used as the capture antibodies. Moreover, it was found that the fluorescence intensity of CD63 and CD9 in the serum EVs of the TB patient group was lower than that in the healthy individuals’ group, which also suggests that although the protein markers of EVs, namely, CD63, CD81, and CD9, are present in almost all EVs [60], the current assay mainly focuses on the overall protein expression content, and the co-expression of each marker in EVs is less frequently reported, which also suggests that the difference in co-expression may be related to the diagnosis or treatment of the disease, which can be explored in greater depth in the future.

Some study authors suggest that the serum EVs of TB patients contain miRNAs that can regulate the immune escape of *Mtb* [61,62], results that differ somewhat from our current experimental results. We speculate that the above discrepancy may be attributed to the fact that the serum EVs of TB patients not only contain the relevant components of host cells but also include the appropriate elements of *Mtb* [63]. When studying the physiological functions of serum EVs in patients with TB, the simultaneous synergistic action of multiple substances may therefore be influenced. Naturally, this variation may also be related to the heterogeneity of individuals in the TB patient group, resulting in different contents of the EVs and thus generating different conclusions. Moreover, we hypothesized that the reason for the different conclusions may be related to the different types of cells used in the studies. In order to address these challenges, the number of sera collected from TB patients should be increased and the contents of the serum EVs extracted from TB patients should be sequenced to enable in-depth exploration in subsequent studies.

The current evolution of EVs related to the bodily fluids of TB patients is mainly focused on the development of markers related to TB detection [64]. The authors of recent studies have identified a variety of markers in different bodily fluids, such as serum, plasma, urine, and pleural fluid, such as LAM [65] and MPT64 [66]; however, little is known about the functions of EVs present in the bodily fluids of TB patients. In this study, we confirmed the presence of EVs in the serum of TB patients and the ability of their EVs to regulate cytokine release, promote macrophage polarization to the M1 type, and inhibit BCG survival in the lungs. The above results suggest to us that TB patients’ serum EVs have powerful physiological functions, and their performance in inhibiting BCG survival shows their significant potential in exploring areas such as the prevention of TB. Serum EVs are derived from the human body and contain richer content, and if they are used as a “drug” related to defensive therapy, they carry components homologous to the human body, meaning that they are safer for use, minimizing the occurrence of rejection reactions.

When serum EVs and BCG were co-stimulated, the immune function of the host was impacted, and the survival rate of BCG was reduced. This finding may be due to the fact that after the serum EVs of TB patients entered into the organs of the mice, some EV components could regulate the activation of the immune system of mice, and when BCG entered the organs of the mice, the BCG components, which were similar or identical to those of TB patients’ serum EVs, were able to rapidly induce adaptive immunity, which then produced a powerful killing effect. This finding also indicates that EVs have a strong ability to transport cell-related components and activate immune functions in cells and even in the organism itself. Examination of TB patients’ serum EVs can also provide new suggestions and solutions for the subsequent development of TB-related prevention plan.

## 5. Conclusions

**Figure 7:**
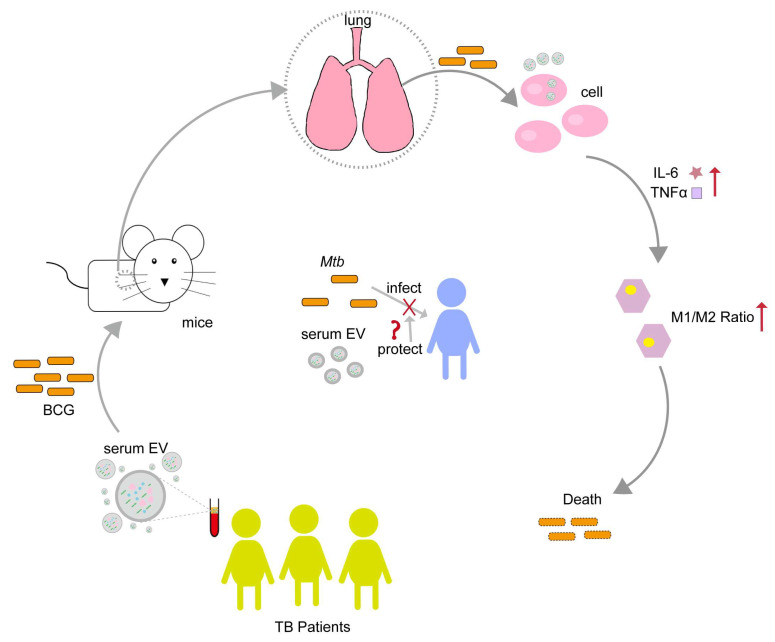
Serum EVs from TB patients have the potential to regulate the immune response against BCG infection. Serum EVs from TB patients stimulated the cellular production of TNF-α and IL-6, modulating macrophage polarization toward the M1 type, and inhibiting BCG colonization of the lung. From this, we speculate that the TB patients’ serum EVs may have some mechanism of "protecting the host" to inhibit *Mtb* infection. Upward red arrows indicate increased expression levels; “❌” indicates inhibition or blocking of *Mtb* infection host; “❓” indicates a protection mechanism may exist.

Based on the above results, we were able to confirm that there were abundant EVs in the serum of healthy individuals and TB patients in our sample and that the serum of TB patients contained fewer EVs co-expressing CD63 and CD9. Serum EVs do not impact cell activity and can be endocytosed by CT26 and RAW264.7. Serum EVs in healthy individuals and TB patients can stimulate the immune cytokines IL-6 and TNF-α secreted by CT26 and RAW264.7, and the activation degree of serum EVs in TB patients is higher. In addition, at the animal level, serum EVs in TB patients can induce an increase in the M1/M2 ratio of macrophages, inhibit the colonization of BCG in the lungs of mice, and reduce the inflammatory response in lung tissue. The above results also indicate that serum EVs in TB patients exert specific functions in regulating the host’s immune response, suggesting that serum EVs in TB patients may have some form of “protective” effect on the host against pathogen infection (Figure 7).

## Data Availability

The data we used are contained within the article.

## Supplementary Materials

The following supporting information can be downloaded at: https://www.mdpi.com/article/10.3390/microorganisms13071524/s1, Figure S1. Schematic diagram of the experimental design used in this study. Figure S2. TB patients’ serum EVs attenuate the inflammatory response of the spleen. Figure S3. TB patients’ serum EVs did not influence the other organs of the mice. Table S1. Sample information. Table S2. q-PCR primers used in this study. Table S3. EV diameter distribution.

## Abbreviations

The following abbreviations are used in this manuscript:

*Mtb*	*Mycobacterium tuberculosis*
TB	Tuberculosis
EVs	Extracellular vesicles
BCG	*Mycobacterium bovis bacillus Calmette Guérin*
IL-6	Interleukin-6
TNF-α	Tumor necrosis factor-α
IL-10	Interleukin-10
CCL1	C-C motif chemokine ligand 1
IL-4	Interleukin-4
CCL22	C-C motif chemokine ligand 22
