# Supplementary material for: Tuberculosis Patients’ Serum Extracellular Vesicles Induce Relevant Immune Responses for Initial Defense Against BCG in Mice
Source: Microorganisms. 2025 Jun 29;13(7):1524. doi: 10.3390/microorganisms13071524 (PMC12298580; doi:10.3390/microorganisms13071524)
Supplement: Supplementary file 1 [file microorganisms-13-01524-s001.zip › microorganisms-3665378-supplementary.pdf]

microorganisms

Supporting Information

Table Contents

Figure S1. Schematic diagram of the experimental design used in this study.
Figure S2. TB patients' serum EVs attenuate the inflammatory response of the spleen.
Figure S3. TB patients' serum EVs did not influence the other organs of the mice.
Table S1. Sample information.
Table S2. q-PCR primers used in this study.
Table S3. EV diameter distribution.

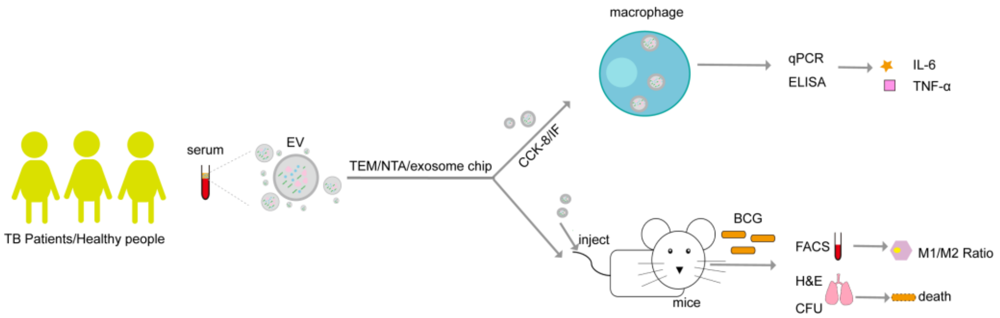

Figure S1. Schematic diagram of the experimental design used in this study.

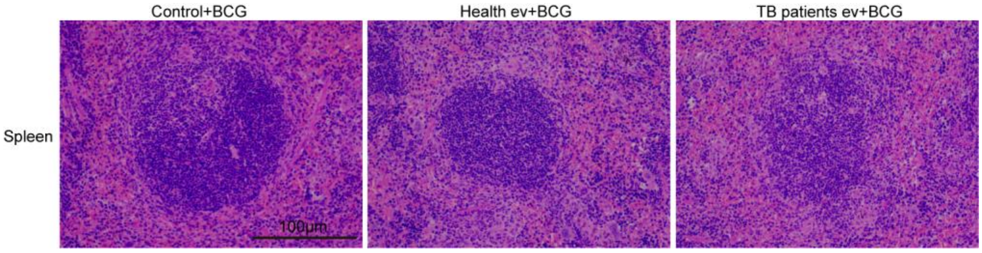

Figure S2. TB patients' serum EVs attenuate the inflammatory response of the spleen. H&E staining was performed on different groups of spleen tissues, and histopathological changes were observed under a microscope. Control + BCG: BCG infected group; Health ev+ BCG: mice stimulated with healthy individuals' serum EVs; TB patients ev + BCG: mice stimulated with TB patients' serum EVs alongside BCG infection. Scale bar: 100 μm.

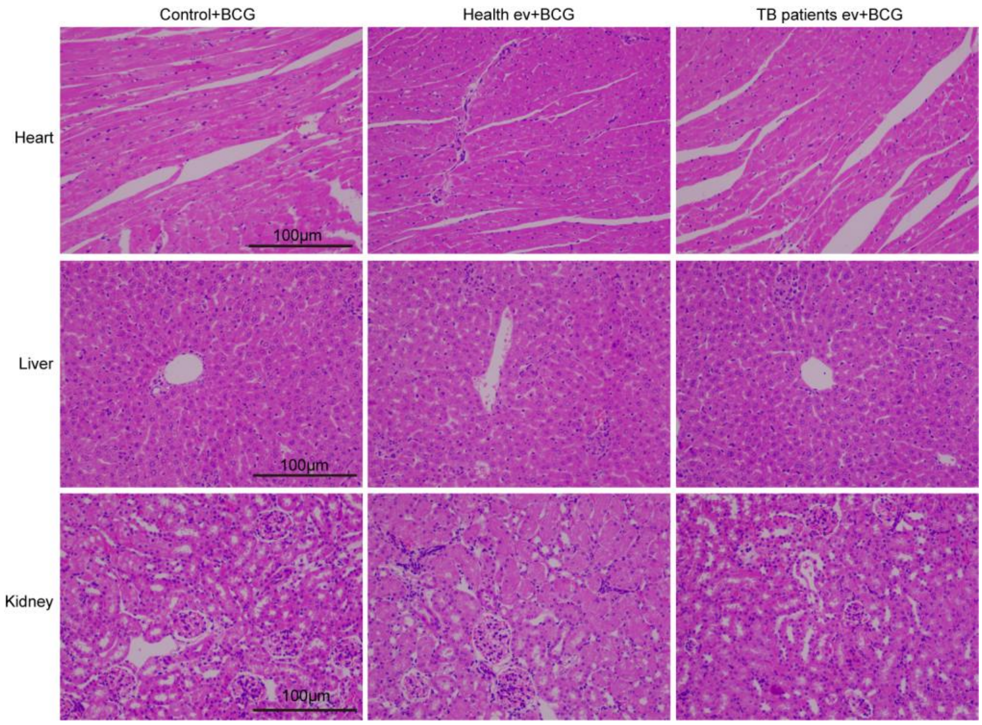

Figure S3. TB patients' serum EVs did not influence the other organs of the mice. H&E staining was performed on different groups of heart, liver, and kidney tissues, and histopathological changes were observed under a microscope. Control + BCG: BCG infected group; Health ev + BCG: mice stimulated with healthy individuals' serum EVs; TB patients' ev + BCG: mice stimulated with TB patients' serum EVs alongside BCG infection. Scale bar: 100 μm.

Table S1. Sample information

Number	Gender	Age	Clinical diagnosis result
1	male	22	TB
2	male	39	TB
3	male	29	TB
4	male	36	Not TB
5	male	69	Not TB
6	male	94	Not TB

Table S2. q-PCR primers used in this study

Name	Sequence (5′-3′)
IL-6 Forward Primer	GAAACCGCTATGAAGTTCCTCTCTG
IL-6 Reverse Primer	TGTTGGGAGTGGTATCCTCTGTGA
TNF-α Forward Primer	GGGTGTTCATCCATTCTC
TNF-α Reverse Primer	GGAAAGCCCATTTGAGT
GAPDH Forward Primer	AGGTCGGTGTGAACGGATTTG
GAPDH Reverse Primer	TGTAGACCATGTAGTTGAGGTCA

The qPCR primer sequences used in this study are shown in the table.

Table S3. EV diameter distribution

Group /nm	Healthy individuals	TB patients
X10	68.2	76.5
X50	109.4	118.8
X90	202.6	217.6
Mean	129.2	136.8
